# *In vitro *cytotoxicity of *Strobilanthes crispus *ethanol extract on hormone dependent human breast adenocarcinoma MCF-7 cell

**DOI:** 10.1186/1472-6882-12-35

**Published:** 2012-04-04

**Authors:** Hueh Zan Chong, Asmah Rahmat, Swee Keong Yeap, Abdah Md Akim, Noorjahan Banu Alitheen, Fauziah Othman, Cheng Lian Gwendoline-Ee

**Affiliations:** 1 Department of Nutrition and Dietetics, Faculty of Medicine and Health Sciences, Universiti Putra Malaysia, Serdang, Selangor 43400, Malaysia; 2 School of Pharmacy and Health Sciences, International Medical University, No. 126, Jalan Jalil Perkasa 19, Bukit Jalil Kuala Lumpur, 57000 Malaysia; 3 Department of Cell and Molecule Biology, Faculty of Biotechnology and Biomolecular Sciences, Universiti Putra Malaysia, Serdang, Selangor 43400, Malaysia; 4 Department of Biomedical Sciences, Faculty of Medicine and Health Sciences, Universiti Putra Malaysia, Serdang, Selangor 43400, Malaysia; 5 Department of Chemistry, Faculty of Science, Universiti Putra Malaysia, Serdang, Selangor 43400, Malaysia

**Keywords:** *Strobilanthes crispus*, MCF-7, Apoptosis, p53

## Abstract

**Background:**

*Strobilanthes crispus *has been traditionally used as antidiabetic, anticancer, diuretic, antilytic and laxative agent. However, cytotoxicity and antiproliferative effect of *S. crispus *is still unclear.

**Results:**

*Strobilanthes cripus *was able to reduce cell viability and proliferation in MTT and BrdU assays. Both cell cycle progression and Tunel assay suggested that IC_50 _of *S. crispus *ethanol extract induced sub-G1 cell cycle phase, and DNA fragmentation. On the other hand, translocation of mitochondria cytochrome c release, induction of caspase 3/7 and p53 while suppress XIAP on treated MCF-7 cell were also observed in this study.

**Conclusion:**

Our findings suggest that *S. crispus *ethanol extract induced apoptosis and DNA fragmentation on hormone dependent breast cancer cell line MCF-7 via mitochondria dependent p53 apoptosis pathway.

## Background

Chemoprevention is a strategy of cancer control by administration of synthetic or natural compounds to intervene in or halt the progress of carcinogenesis. Chemoprevention is a promising anticancer approach aimed to reduce the morbidity and mortality of cancer by delaying the process of carcinogenesis. Chemopreventive plants therefore, provide the genetic basis for cancer therapy [1]. In the last decade, advances in cancer research have enhanced our understanding on the nature of a cancer. Among the most important of these is that genes that control survival and death of cancerous cell. These genes have a major effect on malignancy through the disruption of the apoptotic process that leads to tumor initiation, progression and metastasis. Induction of apoptosis is considered as one of the important targets in chemoprevention. Apoptosis, or programmed cell death, is a cellular suicide program in which individual cells are destroyed while the integrity and architecture of surrounding tissue is preserved [2].

More recently, disease-oriented screening of medicinal plants is utilized as essential tool for new drug discovery. Medicinal plants derived from these studies are being increasingly used by the general public as herbal products on a self-selection basis to either replace or complement modern medicines [3]. One of the medicinal plants that have received great interest among investigators is *Strobilanthes crispus*. This plant is commonly known as 'pecah beling' in Jakarta or 'enyoh kelo'. 'kecibeling' in Java. It is native to countries from Madagascar to Indonesia [4]. Concoctions of *S. cripus *were used as antidiabetic, anticancer, diuretic, antilytic and laxative among traditional practitioners in Malaysia and Indonesia. This plant also has been proven scientifically to possess anti-AIDS, antifungal and antibacterial effects [5]. Previous screening study has reported that methanol extract of *S. crispus *was cytotoxic against HepG2, Caco-2 and MDA-MB-231 while dichloromethane sub-fraction of *S. crispus *possessed cytotoxicity on MCF-7, MDA-MB-231, PC-3 and DU-145 cell via caspase 3/7 [6]. However, cell cycle regulation and details mode of cell death induced by the methanol extract of this plant are still unclear. Therefore, investigation of chemoprevention activities of methanol extract of *S. crispus *towards induction of apoptosis of MCF-7 breast cancer cell was carried out.

## Methodology

### Preparations of the ethanol extract of *Strobilanthes crispus*

Leaves of *Strobilanthes crispus *was collected form herbal garden of Faculty of Medicine and Health Sciences, UPM. The herbarium voucher specimens (No AZ-6803) were identified and deposited by Mr. Ahmed Zainuddin from Department of Botany, Faculty of Science and Technology, Universiti Kebangsaan Malaysia. Leaves of *S. cripus *were then thoroughly rinse with tap and distilled water and were air-dried at room temperature for 2 weeks. Then, the plant samples were homogenized and ground to a fine powder and store in an airtight container. Ground samples of *S. crispus *were soaked 72 h in absolute ethanol for exhaustive solvent extraction. Extracts were filtered with Whatmann paper No 1 and the residues were then re-soaked with a fresh portion of ethanol twice before subjected to evaporation under reduced pressure in a rotary evaporator. The dried residues of plants extracts were resuspended in DMSO (Sigma, USA) for further biological assays.

### Preparation of cell line

Cervical adenocarcinoma tissue HeLa (Cat. No. CCL-2); Hepatocellular carcinoma tissue HepG2 (Cat. No. HB-8065); Colon adenocarcinoma tissues HT-29 (Cat No. HTB-38); Non-hormone dependent breast adenocarcinoma tissues MDA-MB-231 (Cat. No. HTB-26); Hormone dependent breast adenocarcinoma tissues MCF-7 (Cat. No. HTB-22) were obtained from American Type Culture Collection (ATCC, USA). Cell lines were cultured in RPMI 1640 culture media (PAA, USA) containing 10% fetal calf serum (PAA, USA), and 1% of penicillin streptomycin (PAA, USA) in 75 cm^2 ^flask (Nunc, Denmark) at 37°C, 5% CO_2 _environment. Adherent cells at 80% confluence were harvested using Accutase (PAA, USA) for analysis.

### MTT cell viability screening

Cell viability was assessed by measuring the amount of insoluble formazan formed in live cells based on the reduction of 3-(4,5-dimethylthiazol-2-yl)-2,5-diphenyltetrazolium bromide (MTT) salt [7]. Briefly, 100 μl cell suspensions at 1 × 10^5 ^cell/ml were seeded in 96 well microtiter plate (BD, USA). Plant extract in concentration range of 0-100 μg/mL were added. Anthracycline antibiotic Doxorubicin (Sigma, USA) was used as positive control. MTT reagent was added after 72 h exposure followed by dissolution of formed formazan crystal using DMSO (Sigma, USA). Optical density was read with ELISA reader (LX-800) at 550 nm. The dose-response curve is plotted and concentration which gave 50% inhibition of cell growth (IC_50_) is calculated. Concentration that inhibits 50% of cell viability was used as a parameter for cytotoxicity.

### BrdU cell proliferation determination

Cell proliferation was analyzed colorimetrically based on the measurement of pyrimidine analogue BrdU incorporation during DNA synthesis in proliferating cell using BrdU cell proliferation kit (Chemicon, USA). Briefly, cells at 1 × 10^5 ^cell/ml were seeded on 96 well plates (Becton Dickinson, USA). MCF-7 cells were exposed to 30 μg/mL of extract for 24, 48 and 72 h. Ten hours before the end of corresponding period, 10 μl of BrdU labeling solution was added to each well. After exposure of 24, 48 and 72 h, cells were fixed and monoclonal antibody conjugated with peroxidase (POD) was added. Then, TMB (tetramethyl-benzidine) substrate solution was added to allow colour development for photometric detection. Twenty five microlite of sulphuric acid (H_2_SO_4_) 1 M was added to stop reaction before absorbance was read with ELISA reader at 450 nm (reference wavelength: 690 nm).

### Flow cytometry cell cycle progression quantification

After 24, 48 or 72 h of incubation, untreated and *S. crispus *ethanol extract treated (IC_50 _= 30 μg/mL) MCF-7 cells were pelleted and fix in 80% ethanol at -20°C for overnight. After that, the samples were washed twice with 1 ml of PBS, resuspended in 100 μl of RNAse A (200 μg/ml) and incubated for 30 min. Then, 100 μl of propidium iodide (1 mg/ml) was added to the cells and incubated for another 30 min at room temperature. Flow cytometry was performed with a FACS Caliber (BD Biosciences, USA).

### Flow cytometry TUNEL DNA fragmentation analysis

DNA fragmentation of untreated and extract treated MCF-7 cell was tested using TUNEL (terminal deoxynucleotidyltransferase dUTP nick end labeling) assay kit (BD Biosciences, USA). TUNEL-positive cells were apoptotic cells detected with DNA strand breaks which exposed free 3'-hydroxyl ends of double or single stranded DNA fragments labeled with a tracer dUTP. Briefly, after 48 and 72 h of incubation, untreated and *S. crispus *ethanol extract (IC_50 _= 30 μg/mL) treated MCF-7 cells were fixed with 1% (w/v) paraformaldehyde in PBS for 1 h following by incubating with 70% (v/v) ice cold ethanol at -20°C for 30 min. After that, samples were washed with ice cold PBS and added with DNA labeling reagent 1 h before adding dUTP antibody conjugated with FITC (BD Biosciences, USA). Finally, the samples were subjected to flow cytometry analysis using FACS Caliber (BD Biosciences, USA).

### ELISA cytochrome c release and activation of caspases 3/7, 8 and 9 detection

Release of cytochrome c to cytosol (Bender MedSystems, Austria), and activation of caspase 3/7, 8 and 9 (Promega, Madison, WI) was tested using Enzyme Link Immunosorbant Assay (ELISA). Release of cytochrome c was tested on the control and extract treated MCF-7 cell after 12, 18 and 24 h of incubation. On the other hand, activation of caspase 3/7 was tested on 24, 48 and 72 h while caspase 8 and 9 were on 3, 6, 9, 12, 15, 18, 24 and 36 h incubation times. Briefly, both untreated and MCF-7 cells treated with *S. crispus *ethanol extract at IC_50 _concentration (IC_50 _= 30 μg/mL) at the concentration of 10^5 ^were lysed using Triton-X 100 lysis buffer. Cytochrome c, caspases 3/7, 8 or 9 present in the samples was bound to antibodies adsorbed to the surface of the microwells. Fifty microliter biotin-conjugated anti-human cytochrome c, caspases 3/7, 8 or 9 antibodies was added to all wells prior incubation for 2 h at room temperature. Unbound biotin conjugated anti-human cytochrome c antibody was removed during the washing steps. Streptavidin-HRP was added to bind the biotin-conjugated anti-human cytochrome c, caspases 3/7, 8 or 9 antibodies and further incubated for 1 h. Unbound Streptavidin- HRP was removed using wash buffer and 100 μl substrate solution reactive with HRP was added to all wells. Coloured products were formed in proportion to the amount of human cytochrome c present in cells. The reaction is terminated by addition of acid and absorbance is measured at 450 nm. The relative concentration of cytochrome c, caspases 8 and 9 were obtained by comparisons to the plotted their respective standard curve while activity of caspases 3/7 for each respective time point was expressed as the fold change obtained using the following formula:

Absorbanceoftreatment-Absorbanceofcontrol

Foldchangeofcaspase3/7=Absorbanceofcontrol

### Flow cytometry apoptosis and cell cycle regulators protein quantification

Level of cell cycle related cdk2, and cdk4, were tested after 12, and 24 h of incubation while pro/anti-apoptotic related proteins p53 and XIAP were tested on the control and extract treated MCF-7 cell after 24, and 48 h of incubation. Untreated control and *S. crispus *extract (30 μg/ml) treated cells were collected, fixed and permeabilized using cytofix-cytoperm solution (Becton Dickinson, CA, USA) for flow cytometry intracellular protein analysis. After that, the cells were stained with either p53 (Santa Cruz, CA); cdk2 (Cat no. ab6433, Abcam, UK); cdk 4 (Cat no. ab6315, Abcam, UK) and XIAP (Cat no ab26148, Abcam, UK) primary antibodies. Finally, all the cells were washed and stained with 5 μg/10 μL of either FITC goat anti-mouse Ig (BD Biosciences, USA) or FITC goat anti-rabbit Ig (Abcam, USA) and analysed with FACS Caliber (BD Biosciences, USA). Protein levels of cdk2, cdk4, p53 and XIAP for each time point respectively were expressed as the fold change obtained using the following formula:

%ofpositivestainedcellfortreatment-%ofpositivestainedcellforcontrol

Foldchangeofprotein=%ofpositivestainedcellforcontrol

### Statistical analysis

All experiments were assayed in triplicate (n = 3). Data are expressed as means ± S.D. All statistical analyses were performed using Statistical Package for Social Science (SPSS) version 15. Treatment effects were determined using one-way ANOVA post-hoc analysis. A value of p < 0.05 was considered significant unless indicated otherwise.

## Results

### *Strobilanthes crispus *decreased viability and proliferation of MCF-7 cells

**Figure 1:**
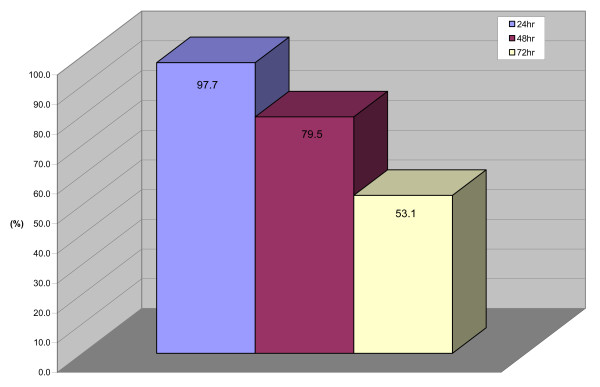
**Effects of *Strobilanthes crispus *extract (IC_50 _concentration = 30 μg/mL) on cell proliferation of MCF-7 breast cancer cell lines**. Cells were seeded in 96-well plates at a density of 10^5 ^and treated for 24, 48 and 72 hr. Values are presented as means (n = 3) ± S.E. *signified statistical difference (p < 0.05).

*Strobilanthes crispus *displayed selective cytotoxicity effects towards various cancer cell lines including cervical, colon, liver and breast cancer cell lines (Table 1). Cytotoxicity activity of *S. crispus *was found to be highly effective against hormone dependent breast cancer cells MCF-7. Anti-proliferative activities of *S. crispus *extract were further investigated in MCF-7 cells. Exposure of *S. crispus *extract at IC_50 _concentration (30 μg/mL) for 24, 48 and 72 h exhibited anti-proliferative effects in MCF-7 cells, portrayed by decrease in the percentage of cells detected with BrdU incorporation (Figure 1). These results suggested that *S. crispus *extracts may exhibit cytotoxic as well as anti-proliferative effects against MCF-7 cells.

**Table 1:** Concentration that inhibits 50% of cell viability (IC_50_) by *Strobilanthes crispus *(SC) extract against selected cell lines after 72 hr incubation

Cell lines	IC_50 _(μg/mL)	
	*Strobilanthes crispus*	Doxorubicin (Sigma, USA)
Cervical adenocarcinoma tissue (HeLa)	78 ± 1.5	NA
Colon adenocarcinoma tissues (HT-29)	52 ± 6.3	52.2 ± 2.9
Non-hormone dependent breast adenocarcinoma tissues (MDA-MB-231)	> 100	4.5 ± 0.1
Hormone dependent breast adenocarcinoma tissues (MCF-7)	30 ± 3.1	2.7 ± 0.2

NA: Not available.

Values are presented as means (n = 3) ± S.E. and * sign signified statistical difference (p < 0.05)

### *Strobilanthes crispus *increased subG1 population and induced DNA fragmentation in MCF-7 cells

**Figure 2:**
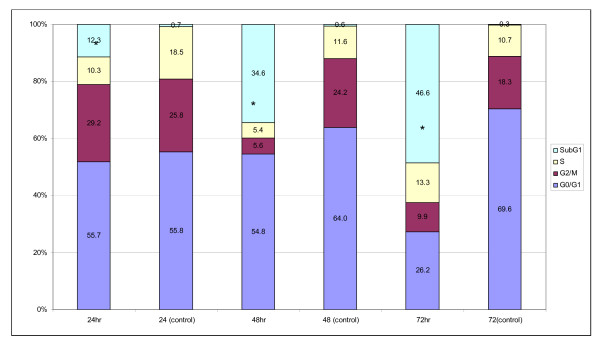
***Strobilanthes crispus *extract (IC_50 _= 30 μg/mL) induced subG1 apoptotic population (%) in treated breast cancer MCF-7 cells after 24 and 48 hr exposure**. Values are presented as means (n = 3) ± S.E. *signified (p < 0.05).

**Figure 3:**
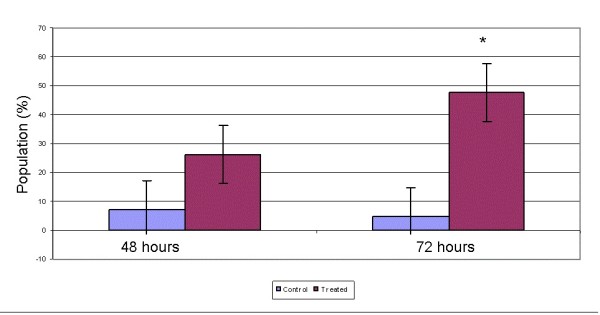
***Strobilanthes crispus *extract (IC_50 _= 30 μg/mL) induced single and double DNA break strands population (%) in treated breast cancer MCF-7 cells after 48 and 72 hr incubation detected by TUNEL assay**. Values are presented as means (n = 3) ± SE. *signified (p < 0.05).

Sub-G1 population was detected in cell cycle analysis for MCF-7 cells treated with *S. crispus *extract. The FACS analyses of cells in control cultures showed 1% of sub-G1 phase population with hypo-diploid DNA as opposed to 12% in the extract treated cells. The sub-G1 phase populations increased significantly upon *S. crispus *extract treatment at 48 and 72 h with 35 and 47% population with hypo-diploid DNA (Figure 2). Besides the cell cycle progression study, flow cytometry TUNEL assay was carried out to confirm the induction of apoptosis by *S. crispus *extract in MCF-7 cells. After 48 and 72 h exposure of *S. crispus *extract, approximate 30 and 50% of MCF-7 cells were stained as TUNEL positive, respectively (Figure 3).

### *Strobilanthes crispus *resulted in translocation of cytochrome c and activation of caspase 3/7 and 9

**Figure 4:**
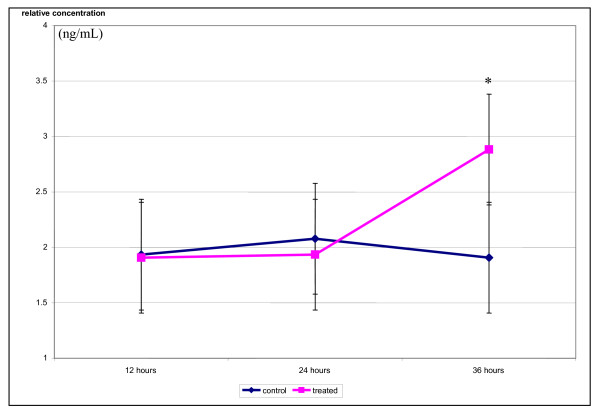
***Strobilanthes crispus *extract (IC_50 _= 30 μg/mL) induced translocation of cytochrome c into the cytosol of treated breast cancer MCF-7 cells**. The relative concentration of cytochrome c was obtained by comparisons to the plotted cytochrome c standard curve. Values are presented as means (n = 3) ± SE. *signified (p < 0.05).

Exposure of *S. crispus *extract resulted in increased relative concentration of cytochrome c in the cytosol of MCF-7 cells. The cytochrome c level in treated MCF-7 cells rose from 24 h (p < 0.05) to 36 hr after the exposure of *S. crispus *extract (Figure 4). Nevertheless, the relative concentration of cytochrome c in control MCF-7 cells remained constant throughout.

**Figure 5:**
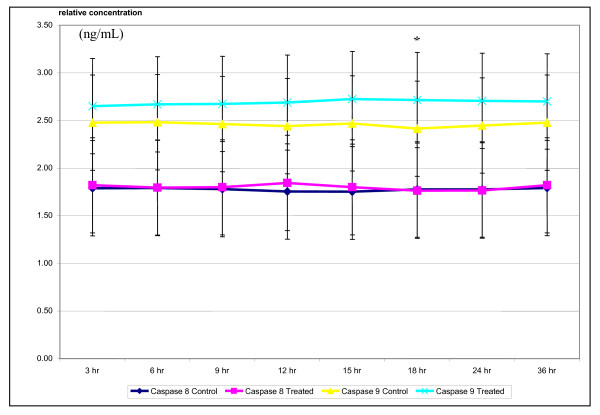
***Strobilanthes crispus *extract (IC_50 _= 30 μg/mL) induced activation of initiator caspase 8 and 9 in treated breast cancer MCF-7 cells**. The relative concentration of caspase 8 and 9 were obtained by comparisons to the plotted caspase 8 and 9 standard curve. Values are presented as means (n = 3) ± S.E. *signified (p < 0.05).

**Figure 6:**
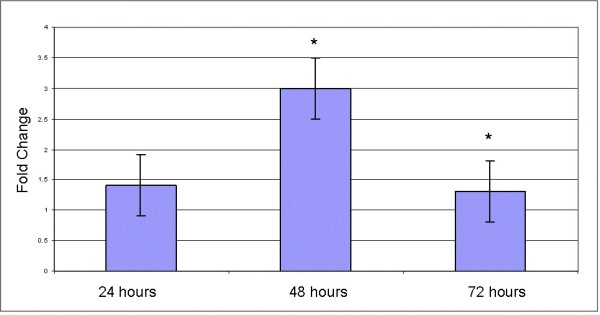
***Strobilanthes crispus *extract (IC_50 _= 30 μg/mL) induced the activity of effector caspase 3/7 (fold change) in treated MCF-7 cells as compared to control cells**. Values are presented as means (n = 3) ± S.E. *signified (p < 0.05).

Increased relative concentration of initiator caspase 9 was observed following exposure of *S. crispus *extract in MCF-7 cells. Although not statistically significant (p > 0.05), steady increase for the level of caspase 9 was still observed after 6 h exposure of the extract. Larger increase in the level of intrinsic pathway initiator caspase 9 was observed at 36 hr of exposure (Figure 5). On the other hand, active caspase 3/7 rose to nearly 3 fold as compared to control after 48 h of exposure to *S. crispus *extract (Figure 6).

### *Strobilanthes crispus *upregulated the expression of tumour suppressor p53 protein while downregulating apoptosis inhibitor protein XIAP in MCF-7 cells

**Figure 7:**
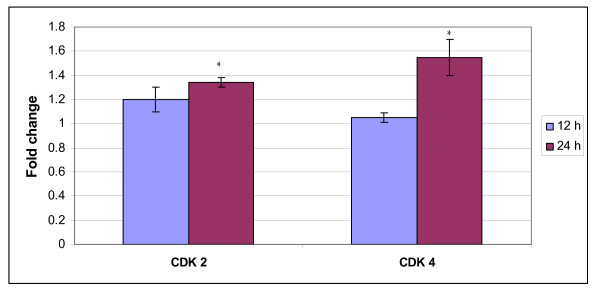
***Strobilanthes crispus *extract (IC_50 _= 30 μg/mL) upregulated the level of cyclin dependent kinase 4 and cyclin dependent kinase 2 (fold change) in treated MCF-7 cells as compared to control cells**. Values are presented as means (n = 3) ± S.E. *signified (p < 0.05).

Induction of mitochrondrial activated apoptosis pathway by *S. crispus *in MCF-7 cells involved modulation of protein level in tumour suppressor p53, cyclin dependent kinase 4, and cyclin dependent kinase 2 protein. The level of cyclin dependent kinase 4 and cyclin dependent kinase 2 levels had statistically increased at 24 h as compared to 12 h in *S. crispus *treated MCF-7 cells as opposed to control MCF-7 cells (Figure 7). Increased in tumour suppressor p53 protein, cyclin dependent kinase 4 and cyclin dependent kinase 2 level were associated in tipping the fate of cells towards cell death rather than cell arrest.

**Figure 8:**
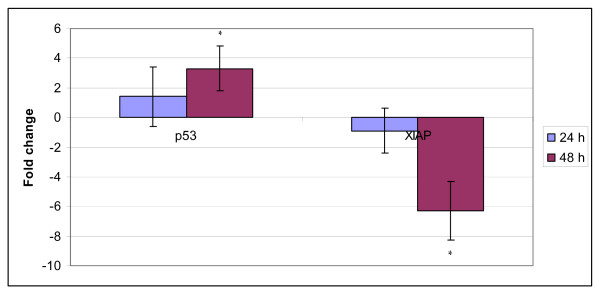
***Strobilanthes crispus *extract (IC_50 _= 30 μg/mL) induced the expression of tumour suppressor p53 protein while downregulated apoptosis inhibitor protein XIAP (fold change) in treated MCF-7 cells as compared to control cells**. Values are presented as means (n = 3) ± S.E. *signified (p < 0.05).

Induction of apoptosis by *S. crispus *extract in MCF-7 cells was found to involve mitochondrial p53 dependent pathway. Exposure of *S. crispus *extract in MCF-7 cells increased the expression of tumour suppressor 53 proteins to nearly three fold as compared to control cells. Meanwhile, six fold decrease in the level of XIAP was observed after 48 h incubation with the extract (Figure 8).

## Discussion

Medicinal plants are able to act through several mechanisms to provide protection against cancer [8]. For instance, medicinal plants blocked the initiation of carcinogenesis by scavenging reactive oxygen species, altered metabolism of pro-carcinogens in favor to excrete reactive metabolites, inhibited carcinogen uptake into cells, enhanced DNA repair, and suppress promotion and progression of neoplastic cells. In this study, screening using MTT and BrdU tests revealed that ethanolic extracts of *Strobilanthes crispus *possessed anti-proliferative and selective cytotoxic activities towards various cancerous cells. IC_50 _value obtained for *S. crispus *against MCF-7 was much lower than the IC_50 _value against MDA-MB-231. This effect was similar to dichloromethane sub-fraction of this plant which possessed better cytotoxicity against MCF-7 than MDA-MB-231 [6].

Degradation and fragmentation of DNA were detected in MCF-7 cells exposed to *S. crispus *extracts in flow cytometry cell cycle RNAse/PI and Tunel assay analysis. In cell cycle study, small fragments of DNA accumulated in the MCF-7 cell were observed as a hypodiploid or 'sub-G1' peak in a DNA histogram and this population indicating the presence of apoptotic population. Present of this sub-G1 population is due to breakage of the linkers between the nucleosomes in the chromatin by endonucleases [9,10]. Added to this, terminal deoxynucleotidyltransferase dUTP nick end labeling (TUNEL) assay was carried out to confirm the DNA fragmentation of the extract treated cell. In TUNEL assay, double and single stranded DNA fragments were detected with traser dUTP. Detection of double and single stranded DNA fragments in TUNEL assay indicated in situ apoptosis cell death. As strands/nicks occurred at a far higher rate in apoptosis than necrosis and taking into consideration the appearance of the sub-G0/G1 population in cell cycle analysis, thus cell death in MCF-7 cells exposed to *S. crispus *extracts was confirmed to be apoptosis [11,12].

At the cytoplasm level, apoptosis event represents a collection of intricate pathways with numerous proteins actively participating in activities from signal transduction, zymogen-type cascade, surgical execution of key cytoskeletal structures and command center DNA within the marked cell. Activation of the mitochondrial-mediated death pathway involve critical step of increasing the membrane permeability. Increased in membrane permeability caused mitochondrial swelling, outer membrane ruptures, and release of pro-apoptotic factors from intermembranous space. Further evidences of apoptosis induction by *S. crispus *extracts towards MCF-7 were assayed in terms of cellular apoptogenic factors release into the cytoplasm.

To determine the involvement of intrinsic/mitochondrial cell death pathway induced by *S. crispus *extracts towards MCF-7, detection of cytochrome c and caspases were carried out. Rise in cytochrome c in the cytoplasm MCF-7 cells treated with *S. crispus *extracts was detected along with the increased in caspases 3/7 activity which is similar to the effect of dichloromethane sub-fraction of this plant [6]. Increased in the concentration of caspase 9, the initiator caspase involves in intrinsic apoptosis pathway, was also recorded in this study (Figure 5). Release of cytochrome c into the cytoplasm via either induction of mitochondrial permeability transition (MPT), transient pore opening or voltage-dependent anion channel is a crucial event in indicating involvement of intrinsic/mitochondrial activated apoptosis [12-16].

Besides diffusion of cytochrome c to the cytosol, other pro-apoptotic factors including apoptosis protease activating factor-1 (Apaf-1), endonuclease-G and apoptosis-inducing factor that located in the mitochondrial inter membrane were also released following permeabilization of outer mitochondrial membrane. Cytochrome c, together with procaspase-9, Apaf-1 and adenosine triphosphate form supramolecular complex-apoptosome which activates caspase-9 through autocatalysis process [17]. The caspase family proteins (cysteine aspartate-specific proteases) consist of initiator caspases, caspase 8, -9, and -10 and effector caspases, caspase 3, -6, and -7. In response to cellular stress, initiator caspases convert the apoptotic signaling to proteolytic activity to a common execution phase [18]. The mitochondrial-activated caspase-9 cleaved procaspase-3 at internal aspartate residues and generated activation of effector caspases via a zymogen-type cascade. Effector caspases-caspase 3, 7 and 10 served as the central executioner of apoptosis machinery, a point of no return for commitment to death [19]. This caspase pathway is regulated via conversion of zymogens to the active form in response to apoptosis stimuli or can be reversely inhibited by inhibitor apoptosis protein (IAP) family [18].

In this study, exposure of *S. crispus *extracts resulted in the decrease level of inhibitor proteins, XIAP in MCF-7 cells. X-linked inhibitor of apoptotic proteases (XIAP) is the best characterized member of the IAP family that able to block apoptosis through binding of its BIR domains to caspase 9 [20]. Thus, repression of the inhibitor of apoptosis protein family (IAPs) emerged as another new strategy against cancer. Thus, the significant decrease in XIAP level in MCF-7 cells by *S. cripus *extracts had permitted the activation of procaspase 9 and effector caspases and therefore execution of apoptosis. Hence, cell death resulted from exposure of *S. crispus *extracts in MCF-7 cells was found to involve mitochondrial activated pathways and caspase 3/7 as executioner of apoptosis.

Tumour suppressor protein p53, a key player in cell death, has been reported to involve in mitochondrial activated apoptosis pathway [21]. In response to multiple death stimuli, p53 translocates to mitochondria. Mitochondrial accumulation of p53 was reported to trigger waves of apoptosis, trancriptive dependent and independent [22]. The role of p53 in the induction of apoptosis by *S. crispus *extracts towards MCF-7 cells was assayed in this study and we found that exposure of *S. crispus *extracts towards MCF-7 had resulted in increased expression of p53 protein (Figure 8). Significant rise in p53 coupled with down-regulation of XIAP protein in treated MCF-7 cells may have been sufficient stimuli for apoptotic activation.

Besides, cyclin dependent kinases (cdks) activity were also important in promoting apoptosis where cdk2, cdk4 and cdk6 were found upregulated in cells undergoing apoptosis [23,24]. Cdk activation occurs downstream of death signal initiation and as a consequence of degradation or caspase-mediated cleavage of negative regulators of cdks. Thus, cleavage of the cdk inhibitors p21 or p27KIP1 protein has been documented in a number of situations in which apoptosis occurs [25-28].

Plant sterols-stigmasterol and sitosterol were among substances which were being isolated and identified in *S. crispus *[29]. Besides an important structural component of plant membranes [30,31] several studies have indicated that certain type of phytosterols may possess anticancer activity. Cytotoxicity of stigmasterol against cancer cells were evidenced towards breast and colon cancer cells [32-34]. Stigmasterol was reported to induce apoptotic of MDA-MB-231 cells via downregulation of oncogenes c-myc and transcriptive factors p53 [32]. Therefore, plant sterols present in *S. crispus *may be among bioactive component which contributed to the initiation of p53 mediated apoptosis pathways in MCF-7 cells.

## Conclusion

Apoptosis induction by *Strobilanthes crispus *was assayed using various methods to portray different apoptotic MCF-7 cell features. Elicit apoptotic MCF-7 cell death was characterized by cell cycle regulation, DNA degradation accompanied by cytochrome c release and caspase activation. This study has added to the knowledge where induction of apoptosis by *S. crispus *extracts towards MCF-7 cells involved upregulation of p53, cdk2 protein and caspase 3/7 besides down-regulation of apoptosis inhibitors XIAP protein.

## Competing interests

The authors declare that they have no competing interests.

## Authors' contributions

HZC conceived the study, carried out the experimentation, acquisition and analysis of data and drafting of the manuscript. SKY assisted with the concept, analysis of data and drafting of the manuscript. AR provided funding. AR, AMA, NBA, FO and CLG-E conceived, designed and supervised the study and revised the manuscript. All authors have read and approved the final manuscript.

## Pre-publication history

The pre-publication history for this paper can be accessed here:

http://www.biomedcentral.com/1472-6882/12/35/prepub
